# Activation of the PD-1/PD-L1 immune checkpoint confers tumor cell chemoresistance associated with increased metastasis

**DOI:** 10.18632/oncotarget.7235

**Published:** 2016-02-07

**Authors:** Madison Black, Ivraym B. Barsoum, Peter Truesdell, Tiziana Cotechini, Shannyn K. Macdonald-Goodfellow, Margaret Petroff, D. Robert Siemens, Madhuri Koti, Andrew W.B. Craig, Charles H. Graham

**Affiliations:** ^1^ Department of Biomedical and Molecular Sciences, Queen's University, Kingston, Ontario, Canada; ^2^ Department of Urology, Queen's University, Kingston, Ontario, Canada; ^3^ Department of Pathobiology and Diagnostic Investigation, Michigan State University, East Lansing, Michigan, USA; ^4^ Cancer Biology and Genetics, Queen's Cancer Research Institute, Kingston, Ontario, Canada

**Keywords:** PD-1, PD-L1, chemoresistance, immune escape, metastasis

## Abstract

The ability of tumor cells to avoid immune destruction (immune escape) as well as their acquired resistance to anti-cancer drugs constitute important barriers to the successful management of cancer. Interaction between the Programmed Death Ligand 1 (PD-L1) on the surface of tumor cells with the Programmed Death-1 (PD-1) receptor on cytotoxic T lymphocytes leads to inactivation of these immune effectors and, consequently, immune escape. Here we show that the PD-1/PD-L1 axis also leads to tumor cell resistance to conventional chemotherapeutic agents. Using a panel of PD-L1-expressing human and mouse breast and prostate cancer cell lines, we found that incubation of breast and prostate cancer cells in the presence of purified recombinant PD-1 resulted in resistance to doxorubicin and docetaxel as determined using clonogenic survival assays. Co-culture with PD-1-expressing Jurkat T cells also promoted chemoresistance and this was prevented by antibody blockade of either PD-L1 or PD-1 or by silencing of the PD-L1 gene. Moreover, inhibition of the PD-1/PD-L1 axis using anti-PD-1 antibody enhanced doxorubicin chemotherapy to inhibit metastasis in a syngeneic mammary orthotopic mouse model of metastatic breast cancer. To further investigate the mechanism of tumor cell survival advantage upon PD-L1 ligation, we show that exposure to rPD-1 promoted ERK and mTOR growth and survival pathways leading to increased cell proliferation. Overall, the findings of this study indicate that combinations of chemotherapy and immune checkpoint blockade may limit chemoresistance and progression to metastatic disease.

## INTRODUCTION

The ability of tumor cells to avoid immune destruction (immune escape), as well as their acquired resistance to anti-cancer drugs, constitute key barriers to the successful management of cancer. An important mechanism of cancer immune escape involves interaction between the Programmed Death 1 (PD-1) receptor on cytotoxic T lymphocytes (CTLs) with the Programmed Death Ligand 1 (PD-L1) on cancer cells or other host immune cells. [1]. The PD-1/PD-L1 axis is one of several “immune checkpoint regulators” that have physiological roles in self-tolerance and in limiting the duration and amplitude of immune responses, primarily through the inhibition of adaptive T cell responses [2]. Tumor cells co-opt the PD-1/PD-L1 mechanism of immune regulation such that activation of this axis results in suppression of anti-tumor adaptive responses through mechanisms involving induction of CTL anergy, exhaustion, apoptosis and decreased cytokine production [1, 3]. In addition to interfering with CTL function, engagement of PD-1 with PD-L1 increases tumor cell resistance to pro-apoptotic signals including those delivered by cytotoxic immune effectors, staurosporin, and Fas ligation [4]; however, the specific mechanism through which this occurs has not been elucidated. Most solid tumours express PD-L1 at various levels and local factors and molecules, such as interferon gamma (IFNγ), stimulate PD-L1 expression [3, 5]. PD-L1 has emerged as a valuable prognostic marker and several studies have correlated PD-L1 expression with tumor infiltrating lymphocytes (TILs) [6], high histological grade [7] and poor overall survival [8]. Current strategies designed to interfere with PD-1/PD-L1 signaling through the use of humanized monoclonal antibodies (*e.g.* Nivolumab) have shown robust clinical responses in patients with heavily-pre-treated advanced cancers such as melanoma, non-small cell lung cancer, and renal cell carcinoma. Furthermore, there is evidence of PD-1/PD-L1-mediated resistance to radiotherapy and anti-CTLA-4 antibody immunotherapy [9], suggesting that PD-1/PD-L1 axis may serve as a pro-survival mechanism for tumour cells. There is evidence that response to PD-1/PD-L1 blockade therapy is at least partly dependent on the levels of tumor PD-L1 protein [10, 11].

Based on the knowledge that PD-L1 expression protects tumor cells from pro-apoptotic agents [12], and that the PD-1/PD-L1 axis is correlated with negative patient outcomes [8], we postulated that the PD-1/PD-L1 axis also contributes to the acquisition of resistance to conventional chemotherapeutic agents. Here we show that the interaction between PD-1 and PD-L1 increases breast and prostate cancer cell resistance to doxorubicin and docetaxel *in vitro* and that inhibition of the PD-1/PD-L1 axis using targeted therapy against PD-1 enhances the effect of conventional chemotherapy to attenuate metastasis in an *in vivo* model of mammary carcinoma.

## RESULTS

### PD-1/PD-L1 interaction increased clonogenic survival in tumor cells following exposure to chemotherapeutic agents

**Figure 1:**
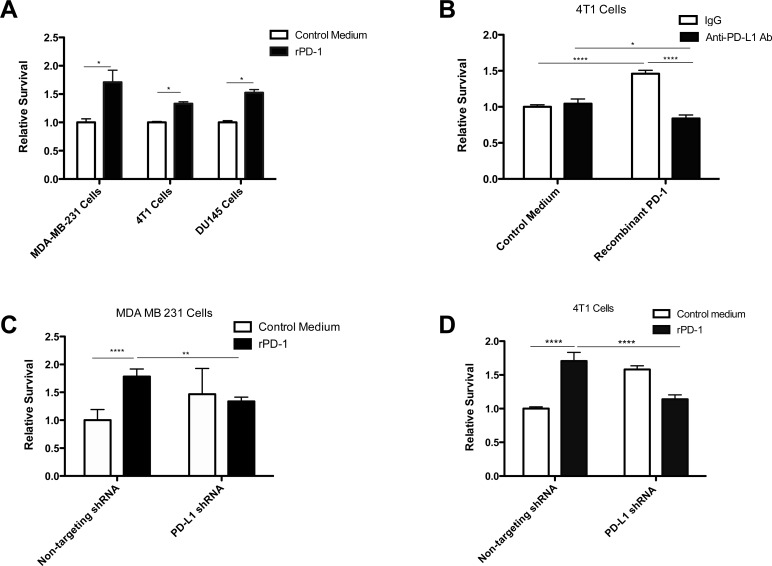
PD-1/PD-L1 interaction results in increased resistance to doxorubicin and docetaxel **A.**, Results of clonogenic assays using MDA-MB-231 cells, 4T1 cells and DU145 cells incubated with recombinant PD-1 (rPD-1; 0.2 μg/ml) for 24 h prior to exposure to doxorubicin (6.25 μM for MDA-MB-231 cells, 2.5 μM 4T1 cells) or docetaxel (1.6 μM DU145 cells). Statistical analysis was performed using an unpaired two-tailed *t*-test. **B.**, Results of clonogenic assays using 4T1 cells incubated with rPD-1 (0.2 μg/ml) for 24 h with or without anti-PD-L1 antibody prior to exposure to doxorubicin (2.5 μM). **C.**, Results of clonogenic assays using MDA-MB-231 cells treated with lentiviral non-targeting or PD-L1-targeting shRNA and incubated with rPD-1 (0.2 μg/ml) prior to doxorubicin exposure (6.25 μM). **D.**, Results of clonogenic assays using 4T1 cells treated with lentiviral non-targeting or PD-L1-targeting shRNA and incubated with rPD-1 (0.2μg/ml) prior to doxorubicin exposure (2.5 μM; left panel). *, *P* < 0.05; **, *P* < 0.01; ***, *P* < 0.0001; ****, *P* < 0.0001. Results of all clonogenic assays are presented as relative survival compared to cells cultured in standard conditions treated with chemotherapy alone. Each graph represents pooled data from at least three independent experiments conducted in replicates of six. Error bars represent the standard error of the mean.

To investigate the contribution of the PD-1/PD-L1 axis to drug resistance in tumor cells we incubated MDA-MB-231, 4T1 and DU145 cells with rPD-1 for 24 h prior to exposure to doxorubicin or docetaxel. We observed increased survival in all cell lines when exposed to rPD-1 prior to doxorubicin (MDA-MB-231 and 4T1 cells) or docetaxel (DU145 cells) (Figure 1A, *p* < 0.05). To assess whether the specific interaction between PD-1 and PD-L1 mediates the observed drug resistance, we blocked PD-L1 using a monoclonal antibody prior to exposure to rPD-1 and subsequent treatment with the chemotherapeutic agent. This resulted in complete inhibition of rPD-1-mediated chemoresistance (Figure 1B, *p* < 0.0001). Furthermore, stable knockdown of PD-L1 expression using human PD-L1-specific or murine PD-L1-specific shRNA prevented the rPD-1-mediated acquisition of resistance to doxorubicin in MDA-MB-231 cells and 4T1 cells (Figure 1C and 1D). Interestingly, MDA-MB-231 and 4T1 cells expressing PD-L1-specific shRNA in the absence of PD-1 were intrinsically more resistant to doxorubicin than their non-targeting shRNA-expressing counterparts. However, the results from the knockdown experiments support the conclusion that the interaction between PD-1 and PD-L1 mediates chemoresistance.

**Figure 2:**
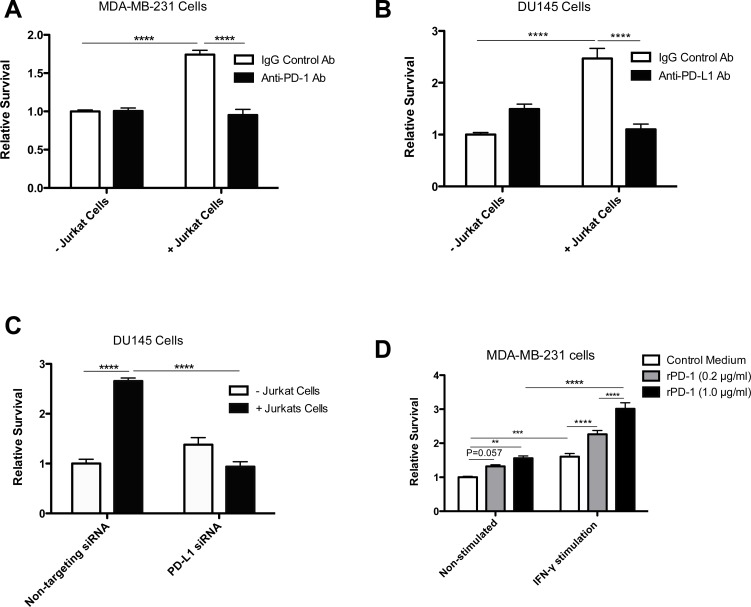
Jurkat T cells increase PD-1/PD-L1-mediated drug resistance in tumor cells **A.**, Results of clonogenic assays using MDA-MB-231 cells incubated with Jurkat T cells (5:1) with or without anti-PD-1 antibody-mediated blockade (1 μg/ml) for 24 h prior to doxorubicin exposure (6.25 μM). **B.**, Results of clonogenic assays using DU145 cells incubated with Jurkat T cells with or without anti-PD-L1 antibody-mediated blockade (4 μg/ml) for 24 h prior to doxorubicin exposure (6.25 μM). **C.**, Results of clonogenic assays with DU145 cells treated with control or PD-L1-targeting siRNA prior to co-incubation with Jurkat T cells for 24 h before treatment with doxorubicin (6.25 μM). **D.**, Survival of MDA-MB-231 cells pre-incubated with or without IFNγ (10 ng/ml) and various concentrations of rPD-1 prior to doxorubicin (12.5 μM) exposure. Data are presented as the mean relative survival compared to cells cultured in standard conditions and treated with doxorubicin alone. *, *P* < 0.05; **, *P* < 0.01; ***, *P* < 0.0001; ****, *P* < 0.0001. Data were pooled from at least three independent experiments conducted in replicates of six. Error bars represent standard error of the mean.

To model a more physiological system, we co-cultured MDA-MB-231 cells or DU145 cells with PD-1-expressing Jurkat T cells [13] for 24 h prior to exposure to doxorubicin. Results from these experiments revealed an increase in drug resistance when tumor cells were exposed to Jurkat cells (Figure 2A, 2B, 2C, *p* < 0.0001). Furthermore, inclusion of blocking anti-PD-L1 or anti-PD-1 antibody (Figure 2A, 2B) or transient knockdown of PD-L1 expression using siRNA (Figure 2C) prevented the T cell-mediated acquisition of resistance to doxorubicin in MDA-MB-231 and DU145 cells.

IFNγ is a known inducer of PD-L1 expression in various cell types, including mammary carcinoma cells [5] and is often present in the tumor microenvironment. To determine whether the extent of PD-1/PD-L1 interactions is associated with the size of the surviving fraction following exposure to anti-cancer drugs we increased the expression of PD-L1 in tumor cells using IFNγ. We then exposed the tumor cells to a low (0.2 μg/ml) or high (1.0 μg/ml) concentration of rPD-1 for 24 h (Figure 2D) and subsequently exposed them to doxorubicin for 1 h. Pre-incubation of cells with IFNγ resulted in increased PD-L1 expression (Supplementary Figure 1A) and enhanced the effect of rPD-1 on resistance to doxorubicin in MDA-MB-231 cells (Figure 2D, *p* < 0.0001). Specifically, cells incubated with IFNγ and exposed to 1 μg/ml of rPD-1 exhibited a 33% increase in survival compared to IFNγ-stimulated cells exposed to 0.2 μg/ml of rPD-1 (*p* < 0.0001). These results provide evidence that the proportion of cells surviving exposure to doxorubicin correlates with the extent of PD-1/PD-L1 interactions.

Interestingly, exposure to IFNγ alone resulted in increased resistance to doxorubicin (Figure 2D, *p* < 0.001). This effect was not limited to MDA-MB-231 cells, as it was also observed when 4T1 and DU145 cells were treated with IFNγ (data not shown).

### Anti-PD-1 antibody treatment combined with doxorubicin resulted in decreased metastasis in mice bearing 4T1 mammary tumors

**Figure 3:**
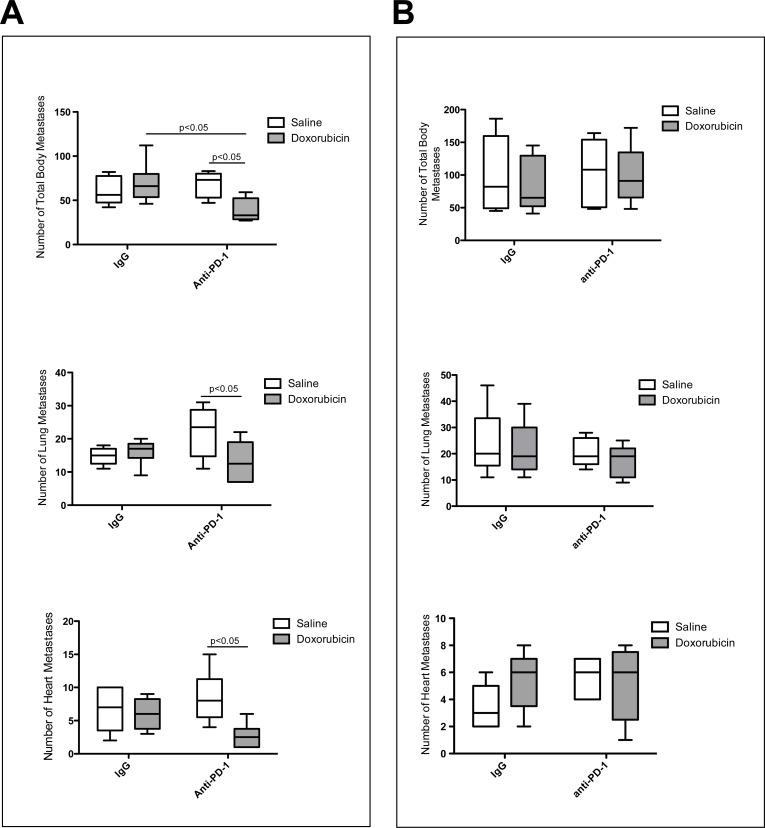
Results from *in vivo* mammary carcinoma studies showing the effect of anti-PD-1 antibody therapy alone or in combination with doxorubicin on 4T1 tumor cell metastasis **A.**, Balb/c Mice treated with combination anti-PD-1 + doxorubicin had significantly fewer total and organ-specific metastases than those receiving either treatment alone. **B.**, Combination anti-PD-1 + doxorubicin therapy had no affect on immunodeficient Rag2^−/−^γc^−/−^ mice. **A.** and **B.**, Total number of metastases analyzed in the lung, liver, spleen, heart, kidney and posterior abdominal wall of Balb/c mice treated with doxorubicin or anti-PD-1 antibody alone or in combination (top) and number of metastases analyzed in the lung (middle) and heart (bottom) of Balb/c mice treated with doxorubicin or anti-PD-1 antibody alone or in combination. Data shown in A are from an experiment consisting of 23 animals, however the experiment was conducted two other times with similar results. Error bars represent standard deviation. **B.**, Total number of metastases in the lung, liver, spleen, heart and kidney of Rag2^−/−^γc^−/−^ mice treated with doxorubicin or anti-PD-1 antibody alone or in combination (top) and the number of metastases analyzed in the lung (middle) and heart (bottom) of Rag2^−/−^γc^−/−^ mice treated with doxorubicin or anti-PD-1 alone or in combination. Data in B were obtained from an experiment consisting of 20 animals. Error bars represent standard deviation.

**Figure 4:**
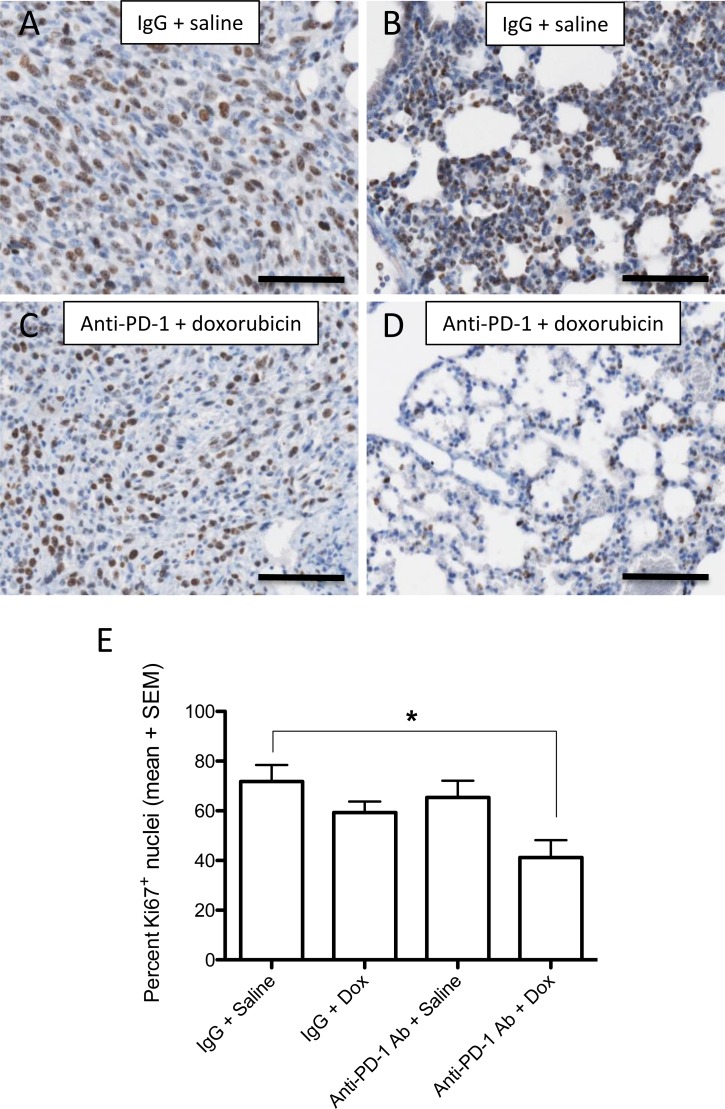
Effect of anti-PD-1 and doxorubicin combination treatment on cell proliferation *in vivo* Immunostaining for Ki67 of primary 4T1 tumors (**A.** and **C.)** and lung metastases (**B.** and **D.)** from mice treated with non-immune IgG + saline **A.** and **B.** or anti-PD-1 antibody + doxorubicin. There was a significant decrease (*P < 0.05) in the percent of Ki67-positive nuclei in lungs from mice treated with combination anti-PD-1 Ab + doxorubicin *versus* lungs from mice treated with control IgG + saline, as determined by ANOVA followed by Bonferroni's *post hoc* test (**E)**. The proportion of Ki67-positive cells in lungs of mice treated with control IgG + doxorubicin or anti-PD-1 antibody + saline was not significantly different from that of untreated (IgG + saline) control mice. Bars = 100 μm.

To determine the role of the PD-1/PD-L1 axis in chemoresistance *in vivo,* we employed an established mouse model of metastatic mammary carcinoma using GFP-tagged 4T1 cells. Mice treated with blocking anti-PD-1 antibody in combination with doxorubicin had a significantly reduced number of metastases (liver, lungs, heart, spleen, kidneys and posterior abdominal wall combined) than those treated with anti-PD-1 antibody or doxorubicin alone (*p* < 0.05; Figure 3A, top). Furthermore, combined treatment with anti-PD-1 antibody + doxorubicin resulted in a significantly reduced number of lung and heart metastases when compared with treatment with doxorubicin alone (*p* < 0.05; Figure 3A, middle & bottom). Interestingly, treatment of mice with anti-PD-1 antibody alone did not have a significant effect on metastasis. Though metastatic lesions were quantified using fluorescence image analysis, histological analysis also revealed that mice receiving combination anti-PD-1 and doxorubicin treatment had fewer Ki67^+^ nuclei when compared to mice that received IgG and saline (Figure 4). To determine if anti-PD-1-antibody-mediated attenuation of chemoresistance was immune-cell specific, a similar experiment was conducted using Rag2^−/−^γc^−/−^ mice deficient in T cells, B cells and NK cells. We did not observe a significant difference in the total number of organ-specific metastases between the treatment groups (Figure 3B). This lack of effect of anti-PD-1 antibody treatment in these immunodeficient mice supports the concept that the chemosensitizing effect of the anti-PD-1 antibody is mediated by immune cells.

Though we did not observe a statistically significant reduction in primary tumor volume in the Balb/c mouse model, combination treatment revealed a trend towards and a smaller final tumor volume (Supplementary Figure 1B, *p* = 0.069) and decreasing tumor growth (Supplementary Figure 2C; *p* = 0.055)

### Exposure to PD-1 increased ERK and mTOR phosphorylation and tumor cell proliferation

**Figure 5:**
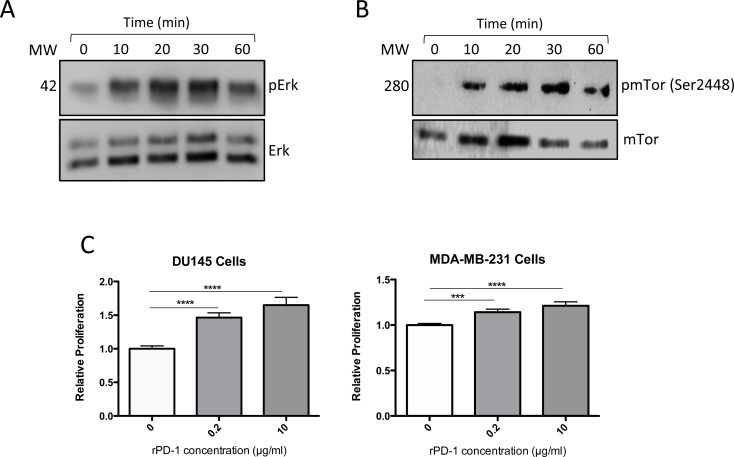
Effect of recombinant PD-1 (rPD-1) on phosphorylated ERK (pERK) and total ERK in MDA-MB-231 cells as determined by Western immunoblot **A.**, Incubation with rPD-1 (10 μg/ml) resulted in almost a two-fold increase in the ratio of pERK : ERK after 30 min in this experiment, with similar results obtained in two additional experiments (Supplementary Table 1). **B.**, Incubation with rPD-1 also increased the ratio of phospho-mTOR (pmTOR) : mTOR in three separate experiments, although to a more variable degree (Supplementary Table 2). In the experiment shown in this figure, the ratio of pmTOR : mTOR peaked at > 100-fold after a 30-min incubation with rPD-1. **C.**, Relative cell proliferation of DU145 or MDA-MB-231 cells following exposure to various concentrations of rPD-1 for 24 hours as determined using the WST proliferation assay in at least three independent experiments conducted in replicates of seven. ***, *P* < 0.001; ****, *P* < 0.0001. Error bars represent standard error of the mean.

To dissect the molecular mechanisms linking PD-1 binding to PD-L1 on tumor cells, we treated MDA-MB-231 cells with rPD-1 and assessed ERK and mTOR survival pathways. Western blot analysis revealed ERK activation that was sustained for up to 60 min (Figure 5A). Similar results were observed for mTOR activation (Figure 5B). Furthermore, exposure to rPD-1 for 24 h resulted in a significant increase in proliferation in both MDA-MB-231 and DU145 cells as determined using a cell proliferation assay (Figure 5C).

## DISCUSSION

This investigation sought to determine the role of the PD-1/PD-L1 axis in the acquisition of resistance to doxorubicin and docetaxel in breast and prostate cancer models, respectively. *In vivo* and *in vitro* findings using human and murine cell lines and two distinct murine models of metastatic breast cancer support the concept that tumor cells may co-opt the host's immune system to acquire resistance to conventional chemotherapeutics. These findings reveal a potential for currently available immune checkpoint blockade approaches specifically designed to interfere with the PD-1/PD-L1 axis to be used as adjuvants to chemotherapy.

A recent publication by Noh *et al.* revealed that co-culture of PD-L1-expressing B16F10 melanoma cells with bone marrow-derived cells results in a smaller proportion of cells undergoing apoptosis/necrosis following gemcitabine treatment [14]; however that study did not assess the contribution of the PD-1/PD-L1 axis specifically. Our study demonstrated that independently blocking PD-1 or PD-L1 resulted in inhibition of PD-1-mediated chemoresistance to a similar extent, and experiments using stable knockdown of PD-L1 expression abolished rPD-1-mediated chemoresistance. The use of distinct human and murine PD-L1 shRNA as well as human PD-L1 siRNA resulting in similar inhibition of PD-1/PD-L1-mediated drug resistance indicates that the effect of PD-L1 knockdown was not gene-construct-specific. Furthermore, using an immunodeficient Rag2^−/−^γc^−/−^ murine model of breast cancer lacking the major PD-1 expressing immune cells (T, B and NK cells), our study showed that blockade of the PD-1/PD-L1 axis using PD-1 blocking antibodies in combination with doxorubicin had no effect on the number of metastases, highlighting the essential role of immune cells in the acquisition of chemoresistance. Due to the immunodeficient nature of the Rag2^−/−^γc^−/−^ mice, tumor progression occurred at a faster rate than in the Balb/c immunocompetent mice and not all mice were able to complete the four cycles of treatment. However, taken together, these findings reveal for the first time that specific PD-1/PD-L1 interactions are at least partly responsible for resistance to doxorubicin and docetaxel. Furthermore, our results indicate that interactions between PD-1 or PD-L1 and other ligands (*e.g.* PD-L2 and B7.1) are unlikely to play a role in the drug resistance phenotype.

Our study demonstrates a novel mechanism by which tumor cells acquire resistance to conventional chemotherapeutics upon engagement of the PD-1/PD-L1 axis and strengthen the rationale for combination chemotherapy/immunotherapy in the clinical setting. Due to the correlation between PD-L1 expression and response to anti-PD-1 treatment [15], most clinical trials are currently focusing on treating cancers known to express high levels of PD-L1 such as melanoma and non-small cell lung cancer [16]. Our *in vivo* data show that although PD-1 blockade or doxorubicin alone did not affect primary tumor growth and metastasis, the combination of these two modalities significantly reduced the overall and organ-specific (lung and heart) number of 4T1 metastases. A lack of effect of PD-1 blockade alone on metastasis using this model may be due to differences in the PD-1/PD-L1 signaling threshold required for drug resistance *versus* immune escape. Alternatively, the synergy observed with combination anti-PD-1 + doxorubicin therapy may be a consequence of chemotherapy-induced immune cell stimulation and tumor antigen release [17]. In the presence of anti-PD-1 antibody, these tumor-specific antigens may be able to promote an effective CTL-mediated anti-tumor immune response.

A study by Azuma *et al.* demonstrated resistance to pro-apoptotic signals in tumor cells following engagement of PD-1 with PD-L1 [4]; however, that study did not reveal a role for major anti-apoptotic and apoptotic pathways in this process. In an effort to determine the mechanism responsible for PD-1/PD-L1-mediated chemoresistance, we explored a potential role of known cell survival molecules. The Ras/Raf/MEK/ERK and RAS/PI3K/PTEN/mTOR signal transduction pathways are well known regulators of cell proliferation and apoptosis which ultimately lead to drug resistance in various cancers including breast [18-20], hematopoietic [21, 22] and liver [23] cancers. Our study revealed a significant increase in ERK and mTOR phosphorylation as well as cellular proliferation in response to rPD-1. These findings indicate that the PD-1/PD-L1 axis may initiate pro-tumorigenic signals leading to chemoresistance. *In vitro* activation of pro-tumorigenic pathways and an increase in cell proliferation following tumor cell exposure to rPD-1 corroborate our *in-vivo* findings showing blockade of PD-1/PD-L1 interaction resulted in reduced Ki67 staining in metastases. Further investigation into how these signals function to promote cell survival is required in order to effectively target PD-1/PD-L1-mediated drug resistance.

The findings of this study indicate that immune checkpoint blockade approaches designed to target the PD-1/PD-L1 axis in cancer patients may be of further therapeutic benefit when used in combination with conventional chemotherapy. The complex tumor microenvironment (TME) composed of immune, stromal and vascular cells and their associated cytokines influence tumor growth and malignant progression. While this study attempted to mimic certain aspects of the TME through the incorporation of T cells and IFNγ, other factors, such as hypoxia could also affect PD-1/PD-L1-mediated chemoresistance. A previous study from our laboratory demonstrated that hypoxia induces PD-L1 expression in tumor cells in a HIF-1α-dependent manner [13]. It is also well known that hypoxia and HIF-1 contribute to the acquisition of drug resistance in cancer cells independently of PD-1 [24, 25]. Therefore, the present study provides evidence of an alternative mechanism by which hypoxia could indirectly contribute to drug resistance in cancer cells. Overall, our findings indicate that, in addition to being an effective immune checkpoint blockade strategy, inhibition of the PD-1/PD-L1 axis may be a novel approach to decrease drug resistance in cancer and, therefore, improve the efficacy of chemotherapy.

## MATERIALS AND METHODS

### Ethics statement

Investigation has been conducted in accordance with the ethical standards and according to the Declaration of Helsinki and according to national and international guidelines and has been approved by the authors' institutional review board.

### Cell lines

Human MDA-MB-231 breast carcinoma cells, DU145 prostate cancer cells and Jurkat T cell leukemia cells were obtained from The American Type Culture Collection (ATCC) and used for the *in vitro* experiments. Mouse 4T1 breast carcinoma cells were obtained from ATCC and used for both *in vitro* and *in vivo* experiments. All cell lines were cultured in RPMI 1640 medium (Invitrogen #11875-119) supplemented with either 10% (MDA-MB-231 cells, 4T1 cells and Jurkat cells) or 5% (DU145 cells) fetal bovine serum (Sigma #F6178). The authenticity of these cell lines has not been verified in the last six months. Cells were maintained under standard culture conditions (37°C in 20% O_2_ and 5% CO_2_).

### Transient and stable PD-L1 knockdown

Transient knockdown of human PD-L1 expression was achieved using Silencer^®^ Select siRNA #s26547 (Ambion Inc.). Silencer^®^ Negative Control siRNA #2 (Ambion Inc.) was used as control. The siRNA (25 nM final concentration) was introduced into cells using siPORT NeoFx reagent (Ambion Inc.) according to the manufacturer's instructions. Treatments with siRNA were performed 24 h prior to incubation in hypoxia or standard conditions.

We used a lentiviral transfection system consisting of GFP-expressing PD-L1 specific short-hairpin RNA (shRNA) (Open Bioscience; mouse #V2LMM_71093, human #V2LHS_53668) to achieve stable PD-L1 knockdown (Supplementary Figure 1). A non-targeting equivalent vector was used as a control (Open Bioscience). Cells that incorporated the cDNA vector were selected in puromycin-containing medium (2-10 μg/ml; Invitrogen #A1113803). PD-L1 knockdown in MDA-MB-231 cells and 4T1 cells was confirmed by immunoblot (R&D Systems #AF156) or flow cytometry (R&D Systems #FAB1019F), respectively (Supplementary Figure 1A and 1B), as described previously [13]. Briefly, 293T cells were transfected with the desired lentiviral plasmid along with psPAx2 (packaging- Gag Pol Rev proteins) and pMD2.g (envelope - VSV-G protein) (Open Bioscience) overnight. Cells were cultured in fresh medium for 48 h before the medium was collected and filtered. Target cells (MDA-MB-231 or 4T1) were seeded in six-well plates at 50,000 cells/well and a dilution series was used to infect the cells. After 48 h, cells underwent puromycin (2-10 μg/ml) selection for 4-5 days. GFP-expression was assessed during the selection period using a UV microscope.

### Stimulation of PD-L1 expression

To stimulate PD-L1 expression, tumour cells were incubated with human IFN*γ* (10 ng/ml; human, eBioscience #14-8319-80) for 24 or 48 h. PD-L1 up-regulation on human MDA-MB-231 cells after incubation with IFN*γ* was confirmed by Western blot analysis as we previously reported [13] (Supplementary Figure 1A).

### Exposure to PD-1

MDA-MB-231, DU145 and 4T1 cells were incubated with recombinant PD-1 (rPD-1; R&D Systems; human: #1086-PD; mouse: #1021-PD) in serum-free medium for 24 h prior to exposure to chemotherapeutic agents and subsequent clonogenic assays. Alternatively, MDA-MB-231 or DU145 cells were co-cultured with human Jurkat T cells for 24 h in a 1:5 ratio. Jurkat cells were stimulated with IL-2 (100U; Sigma, #17908-10KU) prior to co-culture in order to induce activation and stimulate PD-1 expression as previously reported [26]. We have shown that > 90% of Jurkat T cells express PD-1 [13]. In some experiments, we blocked PD-1/PD-L1 interaction with either human anti-PD-L1 antibody (4 μg/ml; Biolegend #329702) or anti-PD-1 antibody (1 μg/ml; R&D Systems #AF1086) prior to exposure to rPD-1 or Jurkat T cells and doxorubicin.

### Clonogenic (colony formation) assay

Clonogenic assays were performed to determine the effect of PD-1/PD-L1 on tumor cell survival following drug exposure as described previously [27]. Tumor cells (50,000 - 100,000) were plated in six-well plates and after various manipulations of the PD-1/PD-L1 axis, were incubated with either doxorubicin (6.25 μM and 12.5 μM for MDA-MB-231; 2.5 μM for 4T1; 6.25 μM for DU145; Sigma #D1515-10MG) or docetaxel (1.6 μM; Sigma #Y0001133) for 1 hr. Drug concentrations were established such that similar surviving fractions were observed across all cell lines and drug types (approximately 0.01). Colony formation assays were performed and scored 7-14 days following plating.

### Immunoblot analysis of survival pathways

For immunoblot analysis, plates of MBA-MB-231 cells stimulated with rPD-1 (1 μg/ml) were rinsed with cold PBS and lysed in RIPA buffer containing the inhibitors leupeptin (10 μg/ml), aprotinin (10 μg/ml), phenylmethylsulfonyl fluoride (1 mM), sodium orthovanadate (1 mM), and sodium fluoride (10 μM). Equal amounts of protein from whole cell lysates were separated by SDS-PAGE and transferred to a PVDF membrane using a Trans-Blot Turbo transfer system (BioRad). Membranes were blocked with 5% skim milk in TBST for one hour at room temperature and incubated overnight at 4°C with the following antibodies: anti-pSer2448 mTor, anti-mTor (Cell Signaling Technology), and anti-pErk1/2 and anti-Erk (Santa Cruz Biotechnology). All antibodies were diluted 1:1000 in TBST. Membranes were washed three times with TBST and incubated with anti-mouse and rabbit HRP-conjugated secondary antibodies (LI-COR), diluted to 1:25,000 in TBST for one hour at room temperature. Protein bands were visualized using enhanced chemiluminescence substrate and quantified using a C-digit Blot Scanner (LI-COR).

### Proliferation assay

The effect of rPD-1 on tumor cell proliferation was assessed using the WST reagent. Briefly, cells cultured in 96-well plates were incubated for 24 h in the absence or presence of rPD-1 (0.2 and 10 μg/ml). Two hours before the end of the incubation period, 10 μl of the WST reagent was added to each well according to the manufacturer's instructions (Roche Diagnostics). Absorbance was measured at 450 nm using an ELISA plate reader.

### Mouse mammary carcinoma models of metastasis

Female Balb/c mice (Charles River) were inoculated with a single orthotopic injection of 3.5 × 10^3^ GFP-expressing 4T1 cells into the lower left mammary fat pad. Once tumours were palpable (about 10 days following tumor cell inoculation), mice were treated twice a week for 15 days with either mouse anti-PD-1 antibody (*n* = 12; 200 μg in 100 μL; Pharmacia Biotech) or control anti-hamster IgG (*n* = 11; Jackson Immunoresearch) delivered intraperitoneally (i.p.). Twenty-four hours after each antibody injection, mice received either doxorubicin (*n* = 12; 5 mg/kg; Sigma #D1515) or the equivalent volume of saline (*n* = 11;) i.p. One mouse did not develop a primary tumour and therefore was excluded from the study. Tumor dimensions were measured after each antibody treatment using digital calipers and volumes were calculated using the formula (length × width)^2^ × 0.5. Overall, mice received four treatment cycles of anti-PD-1 Ab or IgG ± doxorubicin/saline throughout the duration of the experiment. On day 15 (experimental endpoint), mice were euthanized and the primary tumour, heart, lungs, liver, spleen and kidneys were excised and analyzed using a Pan-A-See-Ya Panorama biophotonics imaging system (Lighttools Research) followed by image analysis using ImagePro 6.1 software (Media Cybernetics). Primary tumors and lungs from tumor-bearing Balb/c mice were also fixed in formalin and processed for immunohistochemistry using a rabbit polyclonal anti-Ki67 antibody (Abcam ab15580; 1:1,000) in order to localize proliferating cells. Sections were stained using the Discovery XR Staining System (Ventana Medical Systems, Inc.). Staining was visualized using DAB followed by counterstaining with hematoxylin. Sections were scanned on an Aperio CS digital slide scanner (Leica Biosystems) and images were captured using Aperio Spectrum software (Leica Biosystems).

To determine a potential role of PD-1-expressing lymphocytes in PD-1/PD-L1-mediated chemoresistance *in vivo* we adopted the 4T1 mammary carcinoma model described above using mice lacking T, B, and NK cells. Alymphoid female Rag2^−/−^γc^−/−^ mice (provided by Dr. C. Tayade, Queen's University) were inoculated as above with GFP-expressing 4T1 cells. After tumours were palpable (7-10 days following tumor cell inoculation), mice were treated twice a week for up to 15 days with either mouse anti-PD-1 antibody (*n* = 10) or control anti-hamster IgG (*n* = 10) in combination with doxorubicin or saline as described above. Mice were euthanized upon reaching humane endpoints or on day 15 after receiving four rounds of treatment. Because of their immunodeficient nature, a few mice reached humane endpoints prior to completion of the four rounds of treatment (see Supplementary Table 3 for details). Organ metastases were analyzed as described above.

### Statistical analysis

All statistical analyses were conducted using GraphPad Prism 6 software (GradPad Software, Inc). *In vitro* surviving fraction data and *in vivo* metastasis data were analyzed using Student's *t*-test (when comparing two groups) or by one or two-way ANOVA followed by Tukey's multiple comparison's test when more than two groups were compared, unless otherwise stated. Data were considered significant when *p* < 0.05.

## SUPPLEMENTARY MATERIAL TABLES AND FIGURES


