# Supplementary material for: Activation of the PD-1/PD-L1 immune checkpoint confers tumor cell chemoresistance associated with increased metastasis
Source: Oncotarget. 2016 Feb 7;7(9):10557–67. doi: 10.18632/oncotarget.7235 (PMC4891140; doi:10.18632/oncotarget.7235)
Supplement: Supplementary file 1 [file oncotarget-07-10557-s001.pdf]

Activation of the PD-1/PD-L1 immune checkpoint confers tumor cell chemoresistance associated with increased metastasis

Supplementary Material

Supplementary Table 1. Relative levels of ERK phosphorylation in MDA-MB-231 cells exposed to recombinant PD-1 (1 µg/ml) for up to 60 min. Numbers shown indicate the ratio of phospho-ERK : ERK densitometric values from three independent experiments. Fold increase relative to Time 0 is shown in brackets.

Time (min)	Experiment 1	Experiment 2	Experiment 3
**0**	0.27 (1)	0.42 (1)	0.20 (1)
**10**	0.76 (2.82)	0.44 (1.05)	0.33 (1.83)
**20**	0.75 (2.79)	0.56 (1.33)	0.65 (2.43)
**30**	0.71 (2.66)	0.77 (1.85)	0.83 (2.87)
**60**	0.68 (2.55)	0.67 (1.61)	0.72 (2.58)

Supplementary Table 2. Relative levels of mTOR phosphorylation (Ser2448) in MDA-MB-231 cells exposed to recombinant PD-1 (1 μg/ml) for up to 60 min. Numbers shown indicate the ratio of phospho-mTOR:mTOR densitometric values from three independent experiments. Fold increase relative to Time 0 is shown in brackets.

Time (min)	Experiment 1	Experiment 2	Experiment 3
**0**	0.81<br>(1)	0.48<br>(1)	0.01<br>(1)
**10**	1.24<br>(1.53)	0.62<br>(1.29)	0.26<br>(38.51)
**20**	0.71<br>(0.87)	0.58<br>(1.20)	0.30<br>(43.82)
**30**	0.78<br>(0.96)	0.56<br>(1.17)	0.93<br>(135.39)
**60**	0.76<br>(0.93)	0.42<br>(0.87)	0.65<br>(96.43)

Supplementary Table 3. Rag2$^{-/-}$γc$^{-/-}$ mice treatment data. The table outlines each mouse used in the in-vivo immunodeficient mammary carcinoma study and the type of treatment and number of treatments that each mouse received. A complete study consisted of four cycles of treatment, and each cycle consisted of an antibody injection followed by doxorubicin or saline injection 24 h later.

Mouse Detail	Number of Treatment Cycles
**IgG + Saline**	
Mouse #2	4
Mouse #6	4
Mouse #10	4
Mouse #14	2
Mouse #18	3
Average for group	3.4
**Anti-PD-1 + Saline**	
Mouse #4	4
Mouse #8	2
Mouse #12	2
Mouse #16	3
Mouse #20	1
Average for group	2.4
**IgG + Doxorubicin**	
Mouse #3	3
Mouse #7	4
Mouse #11	1
Mouse #15	1
Mouse #19	4
Average for group	2.6
**Anti-PD-1 + Doxorubicin**	
Mouse #1	4
Mouse #5	4
Mouse #9	1
Mouse #13	2
Mouse #17	4
Average for group	3.0

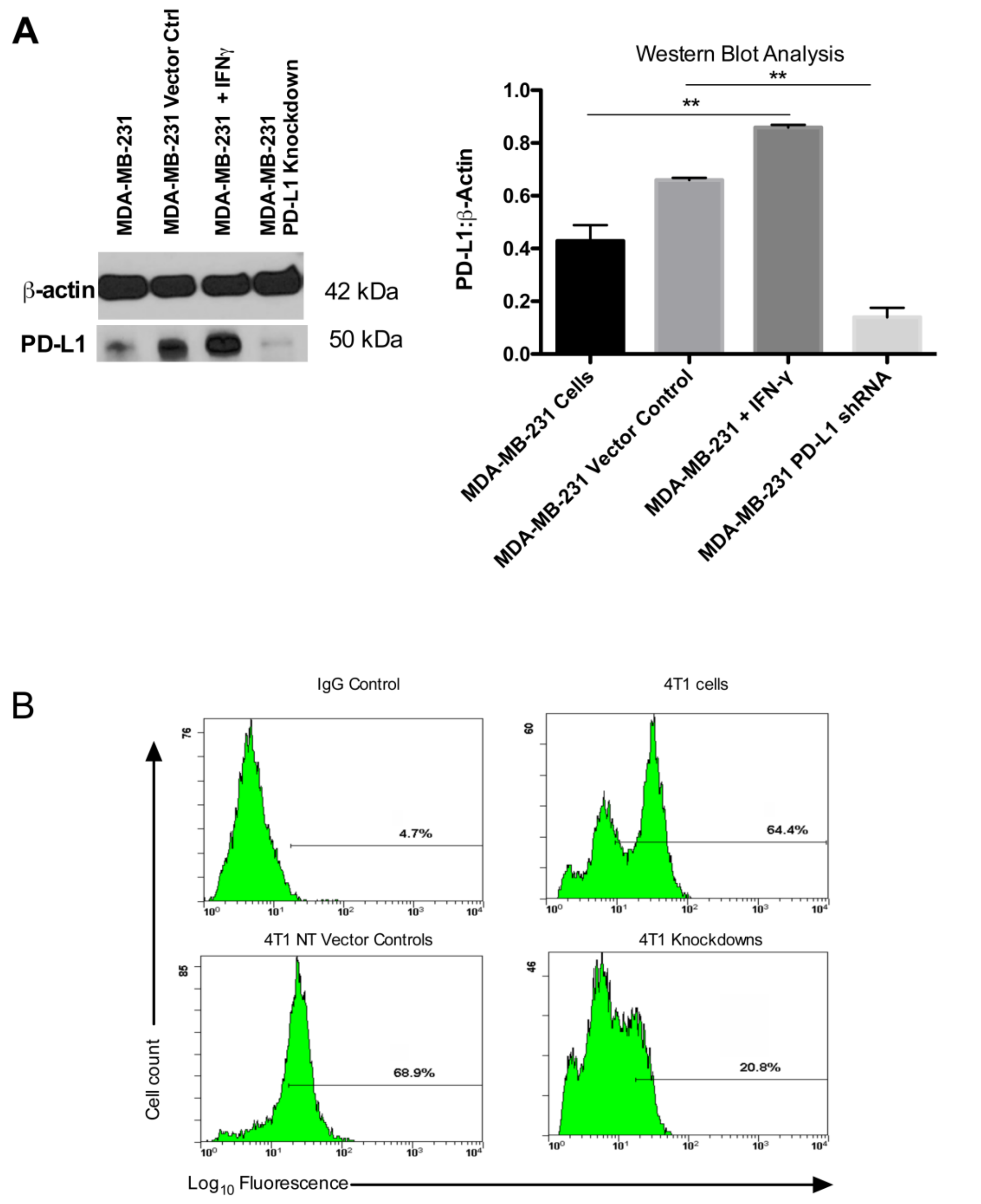

Supplementary Figure 1. PD-L1 knockdown (KD) in MDA-MB-231 and 4T1 cells. A, Western blot representation and quantitative densitometric analysis of PD-L1 protein levels in MDA-MB-231 cells treated under various conditions. Data for densitometric analysis were taken from three independent blots. B, Flow cytometric analysis showing surface PD-L1 expression on 4T1 cells treated with PD-L1 knockdown shRNA or non-

targeting (NT) control vector shRNA and stained with FITC-conjugated IgG control antibody or FITC-conjugated PD-L1 antibody. Following subtraction of background fluorescence of cells incubated with control IgG, 59.7% of control 4T1 cells expressed detectable levels of PD-L1. In contrast, only 16.1% of cells expressing PD-L1 knockdown shRNA (4T1 KD) had detectable surface PD-L1 levels. Compared with cells expressing non-targeting shRNA (4T1 NT Vector Controls; 64.2% of cells with detectable PD-L1) this represents a 75% decrease in surface PD-L1 levels.

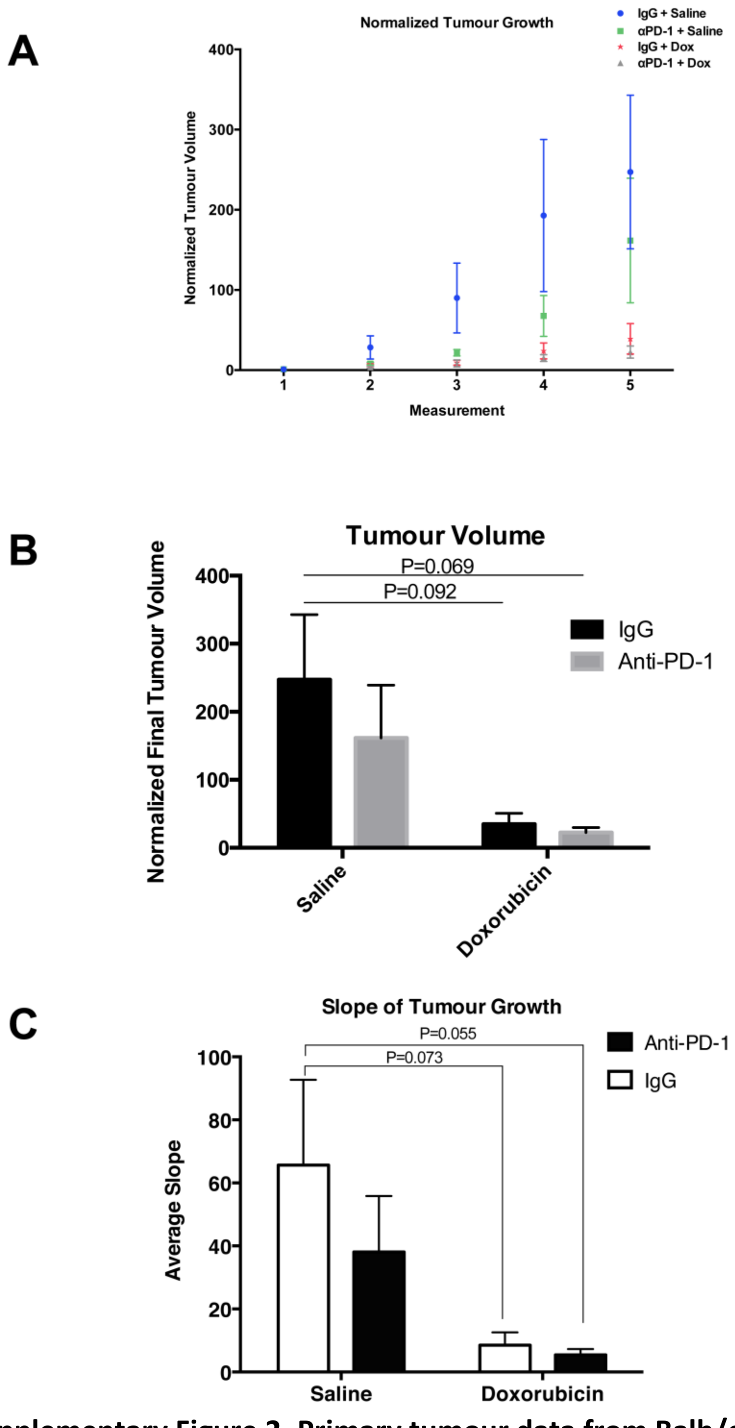

Supplementary Figure 2. Primary tumour data from Balb/c mice treated with anti-PD-1 or doxorubicin in combination or alone. A, Change in mean primary tumor volumes on each day of measurement. Measurements were taken at 2-3-day intervals following each antibody injection and on the day of sacrifice. Tumor volumes for each animal were normalized to the tumour volume on day one of treatment. B, Balb/c mice treated with anti-PD-1 + doxorubicin trended toward having smaller final tumour volumes compared to mice receiving IgG + saline. C, Tumors in mice treated with anti-PD-1 +

doxorubicin exhibited a trend toward a slower growth compared to those in mice receiving IgG + saline as indicated by the differences in the slopes of the growth curves (P=0.055). Data in each graph were obtained from a single experiment consisting of 23 animals, however the experiment was conducted two other times with similar results. Error bars represent standard deviation.
